# Family and Friends: Which Types of Personal Relationships Go Together in a Network?

**DOI:** 10.1007/s11205-015-0987-5

**Published:** 2015-05-19

**Authors:** Jesper Rözer, Gerald Mollenhorst, Anne-Rigt Poortman

**Affiliations:** Department of Sociology/ICS, Utrecht University, Padualaan 14, 3584 CH Utrecht, The Netherlands; Department of Human Geography and Spatial Planning, Utrecht University, Utrecht, The Netherlands; Department of Sociology, Stockholm University, Stockholm, Sweden

**Keywords:** Networks, Personal network, Friendship, Family

## Abstract

We examine the link between family and personal networks. Using arguments about meeting opportunities, competition and social influence, we hypothesise how the presence of specific types of family members (i.e., a partner, children, parents and siblings) and non-family members (i.e., friends, neighbours and colleagues) in the network mutually affect one another. In addition, we propose that—beyond their mere presence—the active role of family members in the network strongly affects the presence of non-family members in the network. Data from the third wave of the Survey on the Social Networks of the Dutch, collected in 2012 and 2013, show that active involvement is of key importance; more than merely having family members present in one’s personal network, the active involvement of specific types of family members in the personal network is associated with having disproportionally more other family members and having somewhat fewer non-family members in the network.

## Introduction

Because almost everyone has a need for personal contacts, it is fortunate that most people in Western societies have one or more (close) personal contacts. Depending on the measurement method used and the examined country, the number of people with one or more close personal contacts ranges from 76 to 98 % of the population (see, e.g., McPherson et al. 2006; Mollenhorst et al. 2008; Schmeets and Te Riele 2014; Wöhler and Hinz 2007). The activities people undertake with these personal contacts, the roles the contacts fulfil in their lives and the support they provide, however, differ among the various types of people in one’s personal network. For example, family members are more likely than friends to provide unconditional support, while friends and other non-family members are more likely to share activities and interests and to also bring people into contact with new ideas (Pahl and Pevalin 2005; Roberts and Dunbar 2011). Consequently, people with a varied personal network may generally have more success with fulfilling their needs for sociability, companionship and support compared with people with a homogenous personal network, who may be more likely to experience feelings of social isolation or a lack of social support or companionship from time to time (Walen and Lachman 2000; Weiss 1974). For these reasons, it is important to gain knowledge about the social composition of personal networks and how different types of relationships may or may not coexist in personal networks.

Existing knowledge about the association between family members and friends in the social network largely stems from research on how family status relates to the size and composition of personal networks (e.g., Bost et al. 2002; Kalmijn 2003, 2012; Munch et al. 1997; Rözer et al. 2014; Song 2012). This research shows, for example, that being married is associated with having more contact with family members and less with friends (Johnson and Leslie 1982; Kalmijn 2003, 2012; Rözer et al. 2014; Song 2012) and that having children is associated with having more contacts with family members and neighbours and less with friends (Kalmijn 2012; Moore 1990; Munch et al. 1997). However, these investigations rarely went beyond people’s family status; consequently, we know far more about how merely having a partner and children relates to the size and composition of people’s personal network than about how the active involvement of specific types of family members and non-family members in the network relates to the involvement of other types of family members and non-family members in the network. In particular, the role of friends and their function in the network compared with that of family members is understudied. One exception is the study of Wrzus et al. (2012); in a study on the well-being of middle-aged and older adults, they found that people with more and closer family relationships reported having fewer friends and vice versa. This supports the idea that the number of friends in the network is associated with the number of family members in the network; as Wrzus and colleagues wrote, they are “linked”.

In this study, we investigate the associations between a broad range of types of family members and non-family members in personal networks. By addressing the presence of various types of family members, we expand upon the family status research tradition. In addition, and more importantly, above and beyond examining how the presence of (specific) personal contacts depends on whether a person *merely has* specific family members, such as a partner and child, we examine how the presence of specific personal contacts in the network depends on the active involvement of these family members in a person’s life. We do so by looking at the influence of family members and non-family members who *fulfil an active role* in one’s personal network. We regard such personal contacts as actively involved in a person’s life because they are the primary contacts with whom an individual undertakes activities and with whom they feel close and intimate. Because of the active involvement of these personal contacts, they may have a great influence on the presence of other close and intimate relationships. Furthermore, we examine how the presence and active involvement of specific types of family members and non-family members are mutually associated. To this end, we distinguish between a partner, children, parents and siblings as family members and friends, neighbours and colleagues as non-family members. Together, these groups form the largest part of people’s personal networks (McPherson et al. 2006; Mollenhorst et al. 2014).

Thus, we examine how the presence and active role of specific types of family members affect the inclusion of other types of family members and of non-family members in personal networks and vice versa. More precisely, we ask the following question: How does the number and presence of (specific types of) personal contacts depend on (a) *having* (specific types of) family members and (b) the *active involvement of* (specific types of) family members and non-family members in the personal network?

To answer these questions empirically, we use recently collected data on personal networks of 947 respondents between 20 and 94 years old and living in the Netherlands. The Netherlands, like other North-western European countries, has reached a high standard of living in the last decades. Although welfare expenditures are declining (or at least are intended to do so), the Netherlands has still one of the most generous welfare states in the world. With public expenditures of 24.3 % of GDP, the Netherlands ranked 12th among the OECD countries in 2013 (OECD 2014). Consequently, citizens generally do not have to rely for support on their social network, and are vice versa less often asked to give extensive help. This creates opportunities to build large social networks based on choice (Van Oorschot and Arts 2005), and might be one of the reasons why the Dutch are less family oriented than citizens of, for instance, Southern European countries (Fokkema et al. 2008). Although the public and political climate regarding ethnic minorities has changed drastically and became increasingly negative towards immigration, immigration levels are still limited in the Netherlands (Van Doorn et al. 2013). About 11.7 of the Dutch Population is immigrant, which is comparable to other European countries such as France (11.6 %), Germany (11.9 %), and England (12.4 %), but somewhat lower than the US (14.3 %) (United Nations 2013). Despite increasing antagonism, compared to citizens in other western countries, citizens in the Netherlands are known to have high levels of (informal) social capital and social trust (c.f. Gesthuizen et al. 2009; Pichler and Wallace 2007). The size of personal networks remained stable over the past decade and is comparable to that in other western countries such as Germany and the United States (Mollenhorst et al. 2014).

## Theoretical Framework and Hypotheses

### Meeting Opportunities, Competition, and Social Influence

The presence and active involvement of specific types of family members and non-family members in personal networks may be associated with each other for at least three reasons: meeting opportunities, competition and social influence. First, the presence and active involvement of family members and friends and of other non-family members in the network may be associated because contact with both types of social contacts is the result of the same meeting opportunity (Feld 1981; Mollenhorst et al. 2008). For example, neighbourhood relationships can emerge among parents whose children play together in their neighbourhood. As a result, having children is positively associated with regarding neighbours as personal contacts.

Second, people may (tacitly) choose to have contact with either family or friends (Homans 1958; Johnson and Leslie 1982). Social contacts are not only capable of fulfilling several needs, such as love, comfort, companionship and information, but also cost several resources, such as time, energy, and cognitive and emotional investments (Saramäki et al. 2014). Therefore, people have to make discriminating choices about whom they consider their personal contacts. For example, people who spend a great deal of time with family members may have less time for friends. Hence, family and friends may be negatively associated. Furthermore, people may prefer to have contact with either family or friends, depending on their life cycle stage. For example, family bonds may be enhanced after the birth of a child, as people choose to embed their child within the family.

Third, from a social influence perspective, important network members may compel one to establish or maintain contact with specific others. For example, one friend might want you to befriend his or her other friends (Heider 1958), or parents may encourage their children to stay close.

### Associations Among Specific Types of Family Members

These three theoretical arguments lead to several general hypotheses about the association between the presence and active involvement of family members and non-family members in personal networks (see Table 1). First, we expect that people who include one family member in their personal network will also include other family members. In other words, family members foster contact with one another. According to the meeting opportunities argument, this may be because when one visits a family member, other family members are often met as well. For example, a person who has a child will often wish to celebrate their child’s birthday with a party to which they invite various family members. Having a large family, and especially having a partner, children and parents who are still alive, increases these meeting opportunities. From this perspective, the mere presence of these family members may often be enough, but the active involvement of family members in one’s personal network (meaning that the individual participates in more activities with family members) increases these meeting opportunities and consequently the likelihood that other family members are or will become part of the personal network.

**Table 1:** Overview of the expected linkage among and between family members and non-family members

	Family	Non-family
Family^a^	Fosters contact (especially within nuclear family)	Restricts contact (especially from partner and child to friends)
Non-family	Restricts contact (especially from friends to partner and child)	No specific effect (besides being possibly self a contact)

^a^Effects of family are expected to be stronger when they are actively involved in people’s personal network instead of when people have them and they may be merely be passively present

Second, according to the competition argument, people need to make discriminating choices about the relationships in which they invest. People who have one family member in their personal network may include other family members as personal contacts because they may be more directed towards their family. For example, people with children may be directed towards their family because they are eager to have their family members involved with their child (and their family members may be eager to see the child) (Moore 1990; Munch et al. 1997).

Third, according to the social influence argument, family members compel each other to remain in contact with other family members, especially those family members with whom they are close themselves. For example, parents may feel at ease when their children have a positive relationship with each other. As a consequence, people who still have a living parent, especially one they regard as a personal contact, are likely to regard any siblings as personal contacts. Likewise, because of their closeness, a partner and children may strongly foster contact with one another.

### Associations Between Specific Types of Family Members and Non-family Members

Whereas relationships with family members in general may foster contact with other family members, they may restrict contact with non-family members. Although, for instance, children may create meeting opportunities in the neighbourhood if they play outside and spouses may introduce their friends to one another, family members and friends are far less likely to provide meeting opportunities for each other compared with family members. According to this theoretical argument, relationships with family members and non-family members are less likely to foster contact with one another.

However, there are many opportunities for competition among relationships with family members and non-family members. Specifically, children and a partner on the one hand and friends on the other hand may compete. Children, a partner, and friends all cost substantial amounts of energy and time but fulfil the same needs for social and emotional support (Laurijssen and Glorieux 2013; Pahl and Pevalin 2005). For example, people may seek intimacy either with their partner or their friends, or they may ask their child or a friend for support (Munch et al. 1997). In addition, the presence of a partner and child encourages people to centre their lives around their family while pushing other relationships towards other circles of intimacy (Ketovski 2012). As a consequence, people who have a partner and/or children, especially when both are considered active personal contacts, may have fewer friends in their personal network. Furthermore, family may claim people’s exclusive intimacy to strengthen intimate family bonds (Ketovski 2012). For example, parents may compel their children to visit their siblings or uncles and aunts instead of visiting ‘just’ some friends. As a side effect, an individual may cut back on their non-familial relations. In addition, partners can become jealous about their spouse’s opposite sex friends, regarding opposite sex friendships as inappropriate and a threat to the romantic relationship (Kalmijn 2002).

Within the non-family part of the network, we do not expect specific effects because of the wide variety of types of non-family social contacts. Friends, neighbours and colleagues all seem to be relatively independent groups. They are often met at different places, seem to fulfil different network functions, and are therefore relatively unlikely to know one another, indeed in particular when neighbours and colleagues are not also considered friends. Hence, it is likely that friends, neighbours and colleagues do not restrict or foster contact with one another.

## Data and Operationalisation

### The Survey on the Social Networks of the Dutch

We use data from the third wave of the Survey on the Social Networks of the Dutch (SSND) (Völker et al. 2013). The third wave covers the most extensive information about respondents’ family situations and the active involvement of family members in their personal networks; therefore, it is suitable for our analyses. Each new wave of the SSND panel dataset is complemented with new respondents to avoid a drain of respondents caused by elusiveness and non-response. In the first wave, conducted in 1999 and 2000, 1007 respondents were interviewed; the respondents represented the Dutch population between 18 and 65 years old. Men were somewhat overrepresented (58 %). In the second wave, conducted in 2007, 998 respondents (604 initial and 394 new) participated, who were between 26 and 72 years old, and 47 % of them was male. In the third wave, collected in 2012 and 2013, 947 respondents participated. Among them were 577 former respondents, of whom 356 respondents had participated in wave 1. In the third wave, respondents where between 20 and 94 years old, of which 50 % was male. Because the original sample has aged and because of sample attrition, compared with basic national socio-demographic characteristics, married, older and more highly educated people are somewhat overrepresented. We controlled for these individual characteristics in the multivariate analyses.

### Measurements

#### Dependent Variable: Inclusion and Number of Personal Network Members

In the third wave of the SSND, 14 ‘name-generating questions’ were used to delineate personal networks. Questions that referred to prescribed relationships at work or in the neighbourhood and questions that refer to soured relationships and online relationships are excluded. As Table 2 shows, this exclusion resulted in five questions that refer to exchange relationships of help and support within the private circle. When answering each of these questions, respondents were allowed to mention network members they had already mentioned in response to previous questions. Additionally, they were allowed to list a maximum number of five names each time.

**Table 2:** Name generating questions to delineate people’s personal network

1	If you are doing an odd job at home and need someone to help you, e.g., to carry furniture or to hold a ladder, who do you ask for help?
2	Many people visit others in their leisure time. Whom do you visit?
3	Do you know people you like going out with to movies, bars, plays, etc. and with whom you actually do this sometimes?
4	Life is not just about going out and having fun. Everybody needs someone to discuss important things with sometimes. With whom have you discussed important personal matters during the past 6 months?
5	Who can you ask for help when you fall ill? Think for example of those who would go to a grocery store or a drugstore for you

After collecting the (nick-)names or initials of personal contacts, name-interpreting questions were asked to gain additional information about the network members. To determine the specific relationship of the network member with the respondent, the questionnaire asked how both were connected. The respondents could choose from 24 categories. These included six types of family members, who could be either be part of the household or not: a partner, parent, child, parent-in-law, sibling and other family member. Non-family members were divided into 12 categories: a friend, boss, immediate colleague, other colleague, former colleague, employee, someone from the neighbourhood, next-door neighbour, former neighbour, co-member of a club or association, acquaintance, and ‘other’. We used these categories to create the dependent variables. First, a distinction was made between the number of *family members* named as personal contacts and the number of *non*-*family members* among the personal contacts. Thereafter, these categories were specified by whether people named at least one *partner, child, parent, sibling,* and *other family member* and *a friend, neighbour, colleague*, and *other non*-*family member*. Table 3 presents descriptive statistics of these and our other variables.

**Table 3:** Descriptive statistics

	Min	Max	Mean	SD
*Personal network*				
Number of family members	0	13	2.75	2.10
Naming a partner	0	1	.56	.50
Naming a child	0	1	.47	.50
Naming a parent	0	1	.17	.38
Naming a sibling	0	1	.35	.48
Naming other family	0	1	.34	.48
Number of non-family members	0	18	3.92	2.71
Naming a friend	0	1	.70	.46
Naming a neighbour	0	1	.67	.47
Naming a colleague	0	1	.20	.40
Naming other non-family	0	1	.19	.39
*Presence of family members*				
Having a partner	0	1	.67	.47
Having a child				
No	0	1	.24	.41
0–12 years of age	0	1	.12	.33
13 year + and lives at home	0	1	.04	.2
Empty nest	0	1	.59	.49
Parents alive				
0	0	1	.60	.52
1	0	1	.21	.41
2	0	1	.19	.39
Number of siblings				
0	0	1	.10	.38
1	0	1	.22	.41
2	0	1	.22	.42
>2	0	1	.46	.50
*Control variables*				
Sex	0	1	.50	.50
Income	0	16	7.26	3.57
Education	0	7	3.99	2.33
Age	20	95	59.27	13.78
Daily activity				
Working	0	1	.45	.50
Pension	0	1	.33	.47
Other	0	1	.22	.42
Denomination				
Catholic	0	1	.16	.37
Protestant	0	1	.20	.40
Other	0	1	.04	.18
None	0	1	.61	.49
Being in good health	0	1	.67	.47

*Source*: SSND3; author’s calculations; *n* = 910

Questions have arisen regarding the reliability of these name-generating and name-interpreting questions. In some other studies, much of the variety in network size and the number of persons who named no contact (social isolates) could be explained by interview effects (Païk and Sanchagrin 2013). Within the third wave of the SSND, however, only 7 out of 910 respondents did not name any person on any of the 5 name-generating questions. As a consequence, only 2.33 % of the variation in the number of isolates could be found at the interview level. A similar figure was found for the number of isolates on the core discussion network; that figure was considerably lower than those found in other surveys, such as over 25 % for some waves of the American General Social Survey (Païk and Sanchagrin 2013) and 40 % for the second wave of the German Pairfam data (Brüderl et al. 2013). Exact binominal probability tests (Païk and Sanchagrin 2013) also showed no notable interviewers. Thus, in an international perspective, the third wave of the SSND may be considered remarkably reliable with respect to interview effects. An explanation for this could be that interviewers were paid per hour instead of per interview and that respondents had become used to the name-generating questions earlier in the interview, making it unlikely for interviewers to skip these questions.

#### Independent Variables: Availability and Inclusion of Family Members and Non-family Members in the Network

As the main independent variable, we distinguished between whether people had access to family members (i.e., whether family members were present in their lives) and whether these family members played an active role in their personal network. We only had information about non-family members if they were actively involved in the personal network. To measure access to specific family members, the respondents were first asked for the number of people with whom they shared a household. Next, they were asked for their relationship to each of these household members, their age, their sex, and some other socio-demographic characteristics. Furthermore, the respondents were asked whether one or both of their parents was still alive and about the number of children, brothers and sisters they had outside their household. Based on all this information, we created binominal variables measuring whether people have *a partner; a child below the age of 12; a child below the age of 18; a child above the age of 18; a child living outside the household who does not mainly live with the respondent’s ex*-*partner (empty nest); parents (alive);* and *siblings*. For the *inclusion of family members and non*-*family members in the personal network,* we employed the network measures described in the previous subsection.

#### Control Variables

In the multivariate analyses, we first controlled for people’s sex because women are generally more directed towards family than men (Moore 1990). Second, we controlled for individuals’ education (8-point scale ranging from not having completed primary education to having a university diploma) and income, because the affluent and more highly educated are known to have larger social networks and to be less directed towards family (McPherson et al. 2006). Income was measured in 17 categories. The first 16 categories increased by 250 euros per category, starting with 1 less than 250 euros a month, to 16 less than 4000 euros a month. The last category is earning more than 4000 euros a month. Furthermore, we controlled for having a paid job, being retired or having another main daily activity to rule out a bias towards groups that have smaller networks or are more directed towards certain types of network members. We controlled for religious denomination (categories: not religious, Protestant, Roman Catholic and other religion) because religious people are known to value family ties more and to have larger networks (Lim and Putnam 2010). To control for the ethnic background of respondents, we include a dummy-coded variable that indicated whether respondents were born in a foreign country (0) or in the Netherlands (1). Almost 10 % of the respondents was not born in the Netherlands. Note that Statistic Netherlands (2014) include the country of origin of people’s parents in their definition of non-natives as well, such that 21.4 % of Dutch society can be counted as immigrants. However, because not all respondents were asked about the country of origin of their parents in the SSND, we rely on information about respondents’ country of origin. Finally, we controlled for age and health status (binomial variable, with (1) indicating good or very good health) because older people and those with poorer mental or physical health are known to have smaller networks and to rely more on family (Carstensen et al. 1999).

### Analytic Strategy

Missing values on all independent variables were imputed with the Mice multiple imputation program for R (Van Buuren and Groothuis-Oudshoorn 2011). There were 17.6 % missing values for income, while the other variables contained less than 5 % missing values. To impute missing data, all relevant information was used. For instance, to impute income, answers to the income questions from previous waves were used. Finally, 50 datasets were created and a maximum of 100 iterations were used.

To test our hypotheses, we first examined the effects of only having certain family members, and second, we examined the effects of including specific types of family members and non-family members in the personal network. We ran negative binominal regression to predict the number of family members and non-family members in the personal network, while logistic regression was used to assess whether specific types of family members (i.e., a partner, children, parents, siblings and others) and non-family members (i.e., friends, neighbours, colleagues, acquaintances and others) were included in the personal network. Binominal variables were created for the specific categories because of the very small number of people who mentioned more than one person in some of these categories (e.g., naming two neighbours or two colleagues). For consistency, we used binominal variables for all of the separate categories.^1^ For each of the models, we only selected respondents who could vary on each dependent variable. For instance, only respondents with at least one child were selected for the models predicting whether people named a child as a personal contact.

## Results

### Descriptive Statistics of the Personal Network

Before testing our hypotheses, we provide the descriptive statistics in Table 4 on the composition of personal networks by presenting the average number of each type of network contact. Almost all of the respondents (99 %) named at least one contact on at least one of the name-generating questions. Respondents named on average 6.67 different personal contacts on the selected five name-generating questions. The largest contribution to these networks came from people they visited (3.61 people on average), while relatively few people were listed who helped with odd jobs around the house (1.95 people).

**Table 4:** Number of personal contacts, by network question and type of contact

Type of contact	Network question					Total Nr.	At least one percentage
	Help at home	Visiting	Going out with	Discussing imp. matters	Help when ill		
Family members	.83	1.35	.83	1.25	1.46	2.75	.88
Partner	.26	.03	.31	.47	.51	.57	.56
Parent	.04	.14	.03	.09	.10	.20	.17
Child	.28	.44	.23	.35	.48	.88	.47
Sibling	.09	.35	.15	.22	.17	.51	.35
Other family members	.16	.39	.12	.12	.20	.59	.34
Non family members	1.12	2.26	1.27	1.07	1.33	3.92	.92
Friend	.17	1.30	.92	.69	.46	1.84	.70
Colleague	.05	.16	.13	.16	.10	.36	.20
Neighbour	.84	.62	.14	.10	.68	1.38	.67
Other	.06	.19	.09	.11	.08	.34	.19
Total	1.95	3.61	2.11	2.33	2.79	6.67	
Naming at least one	.94	.93	.79	.89	.97		.99

The average personal network consisted of 58.8 % non-family members (3.92 people) and 41.2 % family members (2.75 people). Thus, family members played a distinct role in the personal network. Friends play a more important role in having a good time and leisure (visiting, going out), while family members are relatively important for more serious and vital activities (providing help in case of illness, discussing important matters). A partner was named as a personal network member by 56 % of the respondents, but we note that a partner was named by 80 % of those who mentioned having a partner, which is in line with the findings of other studies (McPherson et al. 2006; Wöhler and Hinz 2007). After partners, other family members who were frequently included in the network were children (47 % of the respondents named at least one child), followed by siblings (35 % named at least one sibling). The low figure for parents (only 17 % named at least one parent as a network member) was related to the relatively advanced age of our sample, such that many respondents had no living parent. Of those with at least one living parent, 45 % named at least one parent as a personal network member. Of the non-family members, friends and neighbours were most often included in the personal network (70 % named at least one friend, and 67 named at least one neighbour). Colleagues and acquaintances had a much less prominent position in this part of the personal network (cf. McPherson et al. 2006).

### Multivariate Results

Table 5 shows how the number of family members and non-family members (columns 1 and 7) and the presence of various types of personal contacts (all other columns) in the personal network depend on merely having specific types of family members, such as a partner and children. First of all, we expected that family members fostered contact with one another. This hypothesis was tested in the first six columns. The first column shows that having specific family members, such as a partner, children or siblings, is indeed positively associated with the total number of family members in the network. However, this is primarily because these family members themselves can be included in the personal network. As the second through sixth columns show, little support is found for the hypothesis that merely having specific family members in the network relates to whether more other types of family members will also be in the network. On the contrary, having an adult child is actually related to naming fewer siblings as personal contacts. This relationship could arise because a sibling substitutes for the support a child may otherwise provide. Furthermore, people with older children and more siblings are more likely to consider a child or sibling as a personal contact. This can be explained by the greater support adult children can offer and the greater opportunities to be in good standing with at least one sibling when an individual has multiple siblings.

**Table 5:** Effects of *having specific types of family members* on the number of family members and non-family members (negative binomial regression, results in columns 1 and 7), and on the inclusion of specific types of family members and non-family members (logistic regression, results in columns 2 till 5, and 8 till 10) in the personal network

	Family members						Non-family members				
	Total	Partner	Child	Parent	Sibling	Other	Total	Friend	Neighb.	Col.	Other
	1	2	3	4	5	6	7	8	9	10	11
Intercept	.390	.033	−2.989**	−.423	−.337	−.858	.977**	1.32	−3.205**	−1.449	−1.97~
Having a partner	.397**		−.210	−.142	−.130	.672**	−.049	−.165~	.488*	−.305	−.720**
Child (ref = none)											
0–12 years of age	−.120	−.402		.302	−.265	.455~	−.103~	−.186	.548~	−.214	−.289
13 year—lives at home	.375**	−1.282	1.173*	−.811	.608	.390	−.029	−.386	−.018	−.208	.086
Empty nest	.175**	.026	1.091**	.013	−.412*	−.278	−.051	.029	−.209	.022	.330
Parents alive (ref = 0)											
1	.107	.623~	.438	−.160	−.108	.011	.005	.289	.009	.821**	−.428
2	.067	.672	.118		−.266	−.586*	−.039	.178	−.136	.265	.150
Number of siblings (ref = 0)											
1	.333**	.062	.461	.926		.510	.038	−.768*	.284	.392	−.125
2	.376**	−.327	.434	1.07	.289*	.681*	.028	−.426	.033	.433	−.009
>2	.437**	−.121	.660*	.823	.308*	.418	.094	−.380	.091	.382	.039

*Source*: SSND3

Control variables not shown

* *P* < 0.05, ** *P* < 0.01 (two tailed)

In columns seven through eleven, we tested whether merely having specific types of family members restricts contact with non-family members. Little support is found for this hypothesis. Having a partner is borderline significantly associated with naming fewer friends and also borderline significantly associated with naming fewer ‘other’ non-family members (i.e., non-family members who are not mentioned as a friend, neighbour or colleague). Additionally, those who have more than two siblings are less likely to report a friend as a personal contact. However, in contrast with our hypothesis, we see that people with a partner are more likely to consider their neighbours personal contacts, while those with only one parent alive are more likely to consider a colleague a personal contact.

Results with regard to our control variables (not presented) are generally in line with previous research. Older people are relatively more likely to mention their children and neighbours, and less likely to mention their parents and friends as personal contacts. People who were not born in the Netherlands tend to have smaller networks. This applies to both their family and non-family network. Only minor differences are found with respect to people’s religious denomination, sex, and income.

Table 6 shows how the number of family members and non-family members (columns 1, 2, 8 and 9) and the presence of specific types of personal contacts (all other columns) in the personal network depend on the active involvement of specific types of family members and non-family members in the personal network. Starting with the hypothesis that family members who are mentioned as personal contacts have a larger impact on the inclusion of other network members compared with family members who are not considered personal contacts, we can see more significant and substantially larger effects in Table 6 compared with Table 5. Of course, all models in this table are controlled for the mere passive presence of (specific types of) family members. Hence, as we expected, beyond merely having access to family contacts, the active involvement of family members and non-family members in the personal network is associated with naming other types of personal contacts.

**Table 6:** Effects of *naming (specific types of) family members and non*-*family members as personal contacts* on the number of family members and non-family members (negative binomial regression, results in columns 1,2, 8 and 9), and on the inclusion of specific other types of family members and non-family members (logistic regression, results in columns 3 till 7, and 10 till 13) in the personal network

	Family members							Non-family members					
	Total	Total	Partner	Child	Parent	Sibling	Other	Total	Total	Friend	Neighb.	Col.	Other
	1	2	3	4	5	6	7	8	9	10	11	12	13
Intercept	.434	.355	−.090	−3.743	−.440	−.925	−1.040	1.007**	.995**	1.626	−3.940**	−1.713	−1.160
*Number of family members*								−.028*					
Naming a partner				.915**	.599	−.107	.213		−.080	.222	−.420~	−.563*	−.110
Naming a child			.907**		.642~	.245	.515**		−.100~	.137	−101**	.294	.047
Naming a parent			1.172*	.019		.898**	.795**		−.030	.010	−.341	.567*	.023
Naming a sibling			−.161	.501*	1.027**		.734**		.131**	.134	.540**	.271	−.050
Naming other family			.020	.460*	.806**	.717**			−.080	−.171	−.242	−.262	−.010
*Number of non*-*family members*	−.025*												
Naming a friend		−.022	.485~	.230	.191	.141	−.180				.430*	−.037	−1.060**
Naming a neighbour		−.198**	−.426	−1.154**	−.094	.564**	−.261			.397~		−.083	.421~
Naming a colleague		.071	−.635*	.282	.668*	.286	−.222			−.032	−.080		.502*
Naming other non-family		−.022	−.343	.010	.653	−.010	−.012			−1.069**	.500*	.497*	

*Source*: SSND3

Control variables not shown

* *P* < 0.05, ** *P* < 0.01 (two tailed)

Specifically, we expected the inclusion of specific types of family members to be associated, especially a partner and children (as people from the nuclear family) and parents and siblings (as people from the former nuclear family/parental home). The results shown in Table 6 support these expectations. Naming a partner as a personal contact is positively associated with the inclusion of a child in the personal network (column 4) and vice versa (column 3). Likewise, naming a parent as a personal contact is positively associated with the inclusion of a sibling in the personal network (column 6) and vice versa (column 5). We also show that naming a parent as a personal contact strongly affects the inclusion of one’s partner in the network (column 3), but we found no significant effect the other way around (column 5). Furthermore, naming ‘other’ family members is associated with naming a child, a parent, and a sibling as a personal contact (column 7). Thus, people whose close family members play an active role in their personal network are also more involved with their broader family.

We did not expect to see any clear pattern regarding the effects of including specific types of non-family members on whether other types of non-family members would be included in the personal network. Our results seem to corroborate this expectation. While having a friend as a personal contact is associated with also having a neighbour as a personal contact (column 11), naming a friend as a personal contact is negatively associated with naming at least one ‘other non-family network member’ (column 13) and vice versa (column 10). Furthermore, naming one ‘other non-family network member’ is associated with naming at least one neighbour (column 11) and colleague (column 12) and vice versa (column 13).

Next, we expected that contact with family members would restrict contact with non-family network members and vice versa. The first column of Table 6 shows that as more non-family members are named as personal contacts, fewer family members are included in the personal network. The reverse effect can be observed in column 8. People with one family member as a personal contact have on average .97 times (i.e., exp(−.024)) as many non-family members as personal contacts than people who have no family members in their personal network. This implies that for the average person, each additional family member in the network is associated with naming approximately .10 fewer non-family members and vice versa.^2^ Furthermore, we see that in particular, the inclusion of neighbours in the network is associated with a smaller number of family members in the network (column 2). Specifically, the inclusion of neighbours in the network is negatively associated with naming a child as a personal contact (column 4) and vice versa (column 11). This suggests that children and neighbours are substitutes for one another in personal networks. However, naming a neighbour as a personal contact is positively associated with naming a sibling as a personal contact (column 6) and vice versa (column 11). Additional analyses showed that this is partially because people with siblings in their network simply have a larger network. The inclusion of colleagues in the personal network is negatively associated with naming the romantic partner as a personal contact and vice versa (columns 3 and 12), but it is positively associated with naming a parent as a personal contact and vice versa (columns 5 and 12).

Over and above the inclusion of specific network members, we found few significant effects of merely having specific family members (not presented). In addition, with regard to our other control variables, we found that (a) the elderly are less likely to name their parents and friends as personal contacts, while they are more likely to include their children and friends in the network; (b) women name more friends than men; (c) higher educated respondents name fewer family members and more non-family members; (d) respondents who were born in the Netherlands and those with a better self-rated health reported more social contacts in general; and (e) few significant effects were found for income and denomination.

## Conclusion and Discussion

We investigated the link between the inclusion of family members and non-family members in personal networks. To improve upon previous research, we not only examined the impact of family members who are passively present (e.g., parents who have children) but also looked at the effects of family members and non-family members who play an active role in the respondent’s personal network. More specifically, we studied the link between a broad range of social contacts, including close family, extended family, friends, colleagues and neighbours, while distinguishing between whether people merely have specific types of family members and whether they also name them as personal contacts.

Our findings showed that indeed, beyond the mere presence of family, family members taking an active role in the personal network does have a substantial impact on the inclusion of other personal contacts in the network. This supports our claim that the composition of one’s personal network is not merely determined by family status, life course transitions and family members being around but is more substantially determined by what role or function various types of family members have in the personal network. Therefore, previous research that focused only on the mere presence of certain family members may have underestimated the family’s impact on the composition of an individual’s personal network.

Only when we examined the active involvement of various types of social contacts did we observe clear linkage patterns among and between the inclusion of specific types of family members and non-family members in personal networks. Most of our expectations about these patterns were corroborated. As expected, family members and non-family members—especially a partner and children on the one hand and neighbours and colleagues on the other hand—restrict contact with each other. The more family members that are named, the fewer non-family members that are named as personal contacts and vice versa. This confirmed earlier studies on the linkage between family and non-family (Wrzus et al. 2012). Interestingly, this link was not a zero-sum competition; for an ‘average/model’ person, each additional family member in the personal network is (only) associated with a decrease of approximately .10 Non-family members in the personal network. As a consequence, because the family member is included in the personal network, the network grows to approximately .90 contacts. Similar figures were found for the impact of non-family members on the family part of the network.

Altogether, these findings have several implications. One is that investments in relationships with family members and non-family members can translate into a larger overall network and thus into more options for social support. In particular, children can be seen as investments: when they are young, their presence is likely to decrease the network size, but in later life, they are more likely to expand the network, particularly when they are part of one’s (support) network. Furthermore, we found support for the idea that the inclusion of specific types of family members in the network fosters contact with other family members. Specifically, the inclusion of partners is strongly associated with the inclusion of children in the network and vice versa, while the inclusion of parents is strongly associated with the inclusion of a partner, siblings, and other family members in the network. This indicates that family networks are often dense, which may create feelings of safety and increase the availability of social support. However, those who are primarily focused on their family have slightly less contact with non-family, which reduces the variety in their network and makes them somewhat vulnerable to the dissolution of family bonds (Ketovski 2012).

Future research should investigate more carefully for whom the role of particular types of social contacts is more important and who are more likely to be pulled into their families and to withdraw from other relationships. For instance, the personality, sex, socio-economic status and age of the respondents may play an important role; previous research suggested that the importance of personal network members differs according to these factors (Kahn et al. 2011; Kalmijn 2012; Ketovski 2012; Song 2012; Wrzus et al. 2013). Unfortunately, we were not able to test interactions with these variables because of a lack of power.

Furthermore, given that people can simply forget to mention some personal contacts, even their romantic partner (Mollenhorst et al. 2014), it can be argued that the distinction between the mere passive presence of specific types of family members and their active involvement in the personal network is somewhat arbitrary. However, previous research shows that people are generally more likely to ‘forget’ their weaker and less helpful contacts (Brewer 2000). Moreover, previous research indicated that specific types of associates fulfil specific types of support (Litwak and Szelenyi 1969). Indeed, additional analyses showed that some effects changed where specific questions were concerned. For instance, neighbours were most likely to compete with parents for whom people would ask for help from when they get sick. However, based on the argument that focusing on a specific type of support reduces the reliability of the results, and leads to disregarding the consequences for the larger personal network (Van der Poel 1993), we chose to use the questions simultaneously.

Strictly speaking, we observed cross-sectional relationships between different types of network members, which made it impossible to examine causality and actual influence of existing relationships with specific types of contacts on the emergence of new personal relationships. Fortunately, findings in one of the first large scale longitudinal studies on the influence of existing social contacts on the emergence of new contacts (Kalmijn 2012) were in line with results from cross-sectional data. Nevertheless, more longitudinal studies are necessary to strengthen our confidence in issues of causality.

To conclude, our research clearly indicates that to understand the mutual effects of family status, personal relationships with family members and personal relationships with non-family members, we should not only focus on merely having specific types of family members and non-family members but should also take into account their function in the personal network. Personal networks in modern Western societies are generally composed of both family members and non-family members; family members foster contact with other types of family members, but relationships with family members and non-family members compete only minimally.

## Footnotes

1 Additional analyses in which we treated the specific categories (e.g., a partner, children) as continuous variables did not alter our conclusions. Furthermore, using (zero-inflated) Poisson models to predict the number of family members and non-family members in the personal network did not alter the outcomes either. Marginal differences were observed between men and women and between people of different ages. In all models, the control variables are included but not presented to facilitate comparison between the models.

2 Calculated by subtracting the predicted values for an average person with 1 family/non-family personal contact from the predicted value for an average person without family/non-family personal contacts.

## References

[CR1] Bost KK, Cox MJ, Burchinal MR, Payne C (2002). Structural and supportive changes in couples’ family and friendship networks across the transition to parenthood. Journal of Marriage and Family.

[CR2] Brewer, D. D. (2000). Forgetting in the recall-based elicitation of personal and social networks. *Social Networks, 22*(1), 29–43.

[CR3] Brüderl, J., Huyer-May, B., & Schmiedeberg, C. (2013). Interviewer behavior and the quality of social network data. In P. Winker, P. Rolf, & A. Menold (Eds.), *Interviewers’ deviations in surveys. Impact, reasons, detection and prevention*. Frankfurt: Peter Lang.

[CR4] Carstensen LL, Isaacowitz DM, Charles ST (1999). Taking time seriously—A theory of socioemotional selectivity. American Psychologist.

[CR5] Doorn M, Scheepers P, Dagevos J (2013). Explaining the integration paradox among small immigrant groups in the Netherlands. Journal of International Migration.

[CR6] Feld SL (1981). The focused organization of social ties. American Journal of Sociology.

[CR7] Fokkema T, Ter Bekke S, Dykstra PA (2008). Solidarity between parents and their adult children in Europe.

[CR9] Gesthuizen M, Van der Meer T, Scheepers P (2009). Ethnic diversity and social capital in Europe: Tests of Putnam’s thesis in European countries. Scandinavian Political Studies.

[CR10] Heider F (1958). The psychology of interpersonal relations.

[CR11] Homans GC (1958). Social behavior as exchange. American Journal of Sociology.

[CR12] Johnson MP, Leslie L (1982). Couple involvement and network structure: A test of the dyadic withdrawal hypothesis. Social Psychology Quarterly.

[CR13] Kahn JR, McGill BS, Bianchi SM (2011). Help to family and friends: Are there gender differences at older ages?. Journal of Marriage and Family.

[CR14] Kalmijn M (2002). Sex segregation of friendship networks. Individual and structural determinants of having cross-sex friends. European Sociological Review.

[CR15] Kalmijn M (2003). Shared friendship networks and the life course: An analysis of survey data on married and cohabiting couples. Social Networks.

[CR16] Kalmijn M (2012). Longitudinal analyses of the effects of age, marriage, and parenthood on social contacts and support. Advances in Life Course Research.

[CR17] Ketovski K (2012). The intimate couple, family and the relational organization of close relationships. Sociology.

[CR18] Laurijssen I, Glorieux I (2013). Balancing work and family: A panel analysis of the impact of part-time work on the experience of time pressure. Social Indicators Research.

[CR19] Lim C, Putnam RD (2010). Religion, social networks, and life satisfaction. American Sociological Review.

[CR20] Litwak E, Szelenyi I (1969). Primary group structures and their functions: Kin, neighbours, and friends. American Sociological Review.

[CR21] McPherson M, Smith-Lovin L, Brashears ME (2006). Social isolation in America: Changes in discussion networks over two decades. American Sociological Review.

[CR22] Mollenhorst G, Volker B, Flap H (2014). Changes in personal relationships: How social contexts affect the emergence and discontinuation of personal relationships. Social Networks.

[CR23] Mollenhorst G, Völker B, Flap H (2008). Social contexts and personal relationships: The effect of meeting opportunities on similarity for relationships of different strength. Social Networks.

[CR24] Moore G (1990). Structural determinants of men’s and women’s personal networks. American Sociological Review.

[CR25] Munch A, McPherson JM, Smith-Lovin L (1997). Gender, children, and social contact: The effects of childrearing for men and women. American Sociological Review.

[CR26] OECD. (2014). *National accounts database*. Accessed 6 November 2014. http://stats.oecd.org/Index.aspx?DataSetCode=SOCX_AGG.

[CR27] Pahl R, Pevalin DJ (2005). Between family and friends: A longitudinal study of friendship choice. British Journal of Sociology.

[CR28] Païk A, Sanchagrin K (2013). Social isolation in America: An artifact. American Sociological Review.

[CR29] Pichler F, Wallace C (2007). Patterns of formal and informal social capital in Europe. European Sociological Review.

[CR30] Roberts, S. G. B., & Dunbar, R. I. M. (2011). The costs of family and friends: an 18-month longitudinal study of relationship maintenance and decay. *Evolution and Human Behavior, 32*(3), 186–197.

[CR31] Rözer JJ, Mollenhorst G, Volker B (2014). Romantic relationship formation, maintenance and changes in personal networks. Advances in Life Course Research.

[CR32] Saramäki J, Leicht EA, Lópezb E, Roberts SGB, Reed-Tsochas F, Dunbar RIM (2014). Persistence of social signatures in human communication. PNAS.

[CR33] Schmeets H, Te Riele S (2014). Declining social cohesion in the Netherlands?. Social Indicators Research.

[CR34] Song L (2012). Raising network resources while raising children? Access to social capital by parenthood status, gender, and marital status. Social Networks.

[CR35] United Nations, Department of Economic and Social Affairs, Population Division. (2013). *Trend in international migrant stock: The 2013 revision*. United Nations database, POP/DB/MIG/Stock/Rev.2013.

[CR36] Van Buuren S, Groothuis-Oudshoorn K (2011). Mice. Multivarite imputation by chained equations in R. Journal of Statistical Software.

[CR37] Van der Poel M (1993). Personal networks, a rational-choice explanation of their size and composition.

[CR38] Van Oorschot W, Arts W (2005). The social capital of European welfare states: The crowding out hypothesis revisited. Journal of European Social Policy.

[CR39] Völker B, Schutjens V, Mollenhorst G (2013). The survey on the social networks of the Dutch, third wave (SSND3): Data and codebook.

[CR40] Walen HR, Lachman ME (2000). Social support and strain from partner, family, and friends: Costs and benefits for men and women in adulthood. Journal of Social and Personal Relationships.

[CR41] Weiss, R. S. (1974). The provisions of social relationships. In Z. Rubin & C. Englewood (Eds.), *Doing unto others. Joining, molding, confirming, helping, loving*. London: Prentice Hall.

[CR42] Wöhler, T., & Hinz, T. (2007). Entstehung und entwicklung von sozialkapital. In A. Franze & M. Freitag (Eds.), *Sozialkapital: Grundslagen und anwendungen*. Heusenstamm: Kölner Zeitschrift für Soziologie und Sozialpsychologie.

[CR43] Wrzus C, Wagner J, Neyer FJ (2012). The interdependence of horizontal family relationships and friendships relates to higher well-being. Personal Relationships.

[CR44] Wrzus C, Wagner J, Neyer FJ (2013). Social network changes and life events across the life span: A meta-analysis. Psychological Bulletin.

